# Relationship between Daytime Sleepiness and Intrinsically Photosensitive Retinal Ganglion Cells in Glaucomatous Disease

**DOI:** 10.1155/2016/5317371

**Published:** 2016-02-03

**Authors:** Carolina P. B. Gracitelli, Gloria Liliana Duque-Chica, Ana Laura de Araújo Moura, Marina Roizenblatt, Balazs V. Nagy, Geraldine Ragot de Melo, Paula Delegrego Borba, Sérgio H. Teixeira, Sergio Tufik, Dora Fix Ventura, Augusto Paranhos

**Affiliations:** ^1^Department of Ophthalmology, Federal University of São Paulo, Rua Botucatu, 821 Vila Clementino, 04023-062 São Paulo, SP, Brazil; ^2^Experimental Psychology Department, Institute of Psychology, University of São Paulo, São Paulo, SP, Brazil; ^3^Sleep Medicine Division, Psychobiology Department, Federal University of São Paulo, São Paulo, SP, Brazil

## Abstract

Patients with glaucoma showed to have higher daytime sleepiness measured by Epworth sleepiness scale. In addition, this symptom was associated with pupillary reflex and polysomnography parameters. These ipRGC functions might be impaired in patients with glaucoma, leading to worse quality of life.

## 1. Introduction

The hypothalamic suprachiasmatic nucleus, the primary circadian oscillator in humans, is the site for the master circadian clock [1, 2]. Light phase shifts the endogenous oscillator in the hypothalamic suprachiasmatic nucleus, synchronizing it with the day-night cycle [1, 2]. Some years ago, a study of human eyes culminated in the breakthrough discovery of intrinsically photosensitive retinal ganglion cells (ipRGCs), which constitute a subtype of retinal ganglion cells (RGCs) that express melanopsin, a photopigment that is most sensitive to short-wavelength blue light (480 nm) [3, 4]. In addition to being intrinsically light-sensitive [3, 5–7], ipRGCs are associated with circadian, neuroendocrine, and neurobehavioral functions as well as having influence on some image-forming functions [6–9].

Glaucoma, an optic neuropathy characterized by the progressive loss of RGCs, is associated with morphological changes in the optic nerve and the retinal nerve fiber layer (RNFL) [10]. Several investigators have explored whether light transmission to the hypothalamic suprachiasmatic nucleus or the postillumination pupillary light response (PLR) is compromised as a result of ipRGC damage in glaucoma [11–14]. The activity of ipRGCs has been evaluated in patients with glaucoma using either the light-induced reduction of nocturnal pineal melatonin secretion or the PLR test as a functional indicator of melanopsin-based phototransduction [12–14]. Most of these previous studies have shown significant reductions in ipRGC function in affected eyes compared with the eyes of healthy subjects [11–14]. In addition, previous studies using the PLR test have demonstrated significant correlations between decreased ipRGC function and functional [11, 13] and structural [15] damage in patients with glaucoma.

Few studies have tried to find an association between abnormal ipRGC function and the severity of sleep disorders in glaucoma patients [16, 17]. Wang et al. used the self-rated Pittsburgh Sleep Quality Index (PSQI) to evaluate whether glaucoma affects sleep quality [16]. These authors showed that the prevalence of sleep disorders was higher among patients with glaucoma than among healthy participants [16]. Although these studies indicate that patients with glaucoma exhibit significantly altered sleep patterns that are also correlated with the PLR test, the relationships between objective polysomnographic measures and self-reported diurnal symptoms (e.g., excessive daytime sleepiness) have yet to be established. Furthermore, daytime sleepiness has not been associated with ipRGC functions such as PLR.

The degree of daytime sleepiness can be assessed using the Epworth sleepiness scale (ESS), which can be helpful in diagnosing sleep disorders [18]. The ESS score has never been determined in a glaucomatous population, nor has it been associated with the objective polysomnography measures that are considered as the gold-standard test for assessing sleep disorders. The study uses the PLR (as a parameter for ipRGC dysfunction) and correlated these measurements with ESS and polysomnography to evaluated correlations between sleep disorder and ipRGC function.

## 2. Methods

The protocol for this cross-sectional study followed the tenets of the Declaration of Helsinki and was approved by the Institutional Review Board of the Federal University of São Paulo (CEP 262.470). Written informed consent was obtained from all subjects.

### 2.1. Study Sample

A total of 40 participants were recruited from the Federal University of São Paulo. Thirty of these participants had primary open-angle glaucoma, and 10 others were included for the control group. The inclusion and exclusion criteria, as well as the ophthalmological assessments, have been previously used and are described in detail as follows [15, 17]. All subjects underwent a complete ophthalmological examination, including a medical history review, best-corrected visual acuity measurement, slit-lamp biomicroscopy, gonioscopy, intraocular pressure (IOP) measurement, dilated fundoscopic examination using a 78-diopter (D) lens, refraction, and standard automated perimetry (SAP) using the Swedish interactive threshold algorithm (SITA Standard 24-2; Carl Zeiss Meditec, Inc., Dublin, CA, USA). Subjects were excluded if they were younger than 40 years old or older than 80 years old; had a best-corrected visual acuity worse than 0.2 logMAR (logarithm of the minimum angle of resolution); had a previous history of ophthalmic surgery; had lens opacity greater than 0.5 (cortical opacity, nuclear opalescence, posterior subcapsular opacity, or changes in nuclear color) according to the Lens Opacity Classification System III (LOCS III) [19]; had any corneal, retinal, or orbital diseases; presented with an absolute spherical refractive error >5 D or an absolute cylindrical error >3 D; or were using alpha-adrenergic agonist eye drop medication or any systemic medication that might affect PLR. Participants with a history of taking psychoactive agents were excluded from the study. In addition, none of our patients reported any travel or shift work during the examination period. Only patients with an open angle at gonioscopy were included in our study.

The participants were considered as having glaucoma if they had at least 3 repeatable, consecutive, abnormal visual field test results, which were defined as a pattern standard deviation outside the 95% confidence interval for the normal range or a glaucoma hemifield test result outside the normal limits and a corresponding alteration in the appearance of the optic disc. Patients were also considered as having glaucoma if they showed signs of glaucomatous optic neuropathy at clinical examination by a glaucoma expert, confirmed by stereophotography. Glaucomatous damage to the optic disc nerve was defined as the presence of RNFL defects or localized or diffuse neuroretinal rim loss.

The control participants showed normal results at ophthalmological examination, an IOP of <21 mmHg, normal visual field test results, and absence of glaucomatous optic neuropathy based on fundoscopic and stereoscopic optic disc photograph evaluations.

The entire cohort (i.e., the 30 patients with primary open-angle glaucoma and the 10 controls) belongs to the same sample of a study that was published elsewhere [15, 17]. No additional participant was recruited to augment that previously published sample [15, 17].

### 2.2. Sleep Disorder Questionnaire

Sleep disorders were evaluated using the ESS, which has been widely used as a subjective measurement of daytime sleepiness [18]. This test measures the probability of falling asleep across eight daily situations; each question is scored as 0 (e.g., would never doze), 1 (slight change of dozing), 2 (moderate chance of dozing), or 3 (high chance of dozing), for a total possible score of 24. The clinically normal score of this scale ranges from 2 to 10. A score higher than 10 indicates a borderline or increased level of chronic sleepiness, and a score higher than 15 is defined as excessive daytime sleepiness [18].

### 2.3. PLR Test

Measurement of the PLR was based on a method developed by Park et al. [20]; our previous studies [15, 17] and other research groups have used this technique [21]. To preferentially stimulate ipRGC function, we used 470 nm (blue) flashes with luminance of 250 cd/m^2^ (14.1 log photons/cm^2^/s melanopic excitation) [2] of 1 s in duration. Alternatively, to preferentially stimulate different retinal photoreceptors (e.g., cones and rods) without directly stimulating the ipRGCs, we used 640 nm (red) flashes of 1 s in duration with luminance of 250 cd/m^2^ (10.7 log photons/cm^2^/s melanopic excitation) [2].  Table 1 summarizes full equations for calculating illuminance values for ipRGC, cones, and rods functions [2]. For each stimulus, a red flash was presented first, followed by a blue flash 60 s after the offset of the red flash. The interval between stimuli was used to allow the pupil size to return to baseline before the presentation of the subsequent stimulus. Both eyes were tested monocularly in a random order. One pupillometric measurement per participant was performed. The patients were dark-adapted for 10 min; then, alternating 1 s red and blue flashes were presented at luminance of 250 cd/m^2^ (10.7 and 14.1 log photons/cm^2^/s melanopic excitation, resp.) [2].

Stimuli were generated by corresponding light-emitting diodes (LEDs) in a Ganzfeld system (RETIport; Roland Consult, Brandenburg, Germany), and responses were recorded using an eye-tracking camera system with an infrared LED (IR333-A; Arrington Research, Scottsdale, AZ, USA). The Arrington Research infrared camera recorded the procedure at 60 frames per second. The peak wavelength of the infrared LEDs was 940 nm [20, 22, 23]. Pupil size was directly measured; the largest diameter was considered as the primary measure. The peak PLR amplitude was calculated as the maximal pupillary constriction and expressed relative to the baseline value (peak PLR amplitude = maximal constriction diameter/baseline diameter) [20]. In addition, the sustained PLR was expressed as the pupil diameter 6 s after the flash offset relative to baseline [20]. The absolute values of the peak and sustained responses to blue and red flashes at 250 cd/m^2^ for a similar cohort were reported in our previous studies [15, 17].

### 2.4. Polysomnography

To obtain sleep parameter data, polysomnographic recordings were performed over a full night in a temperature-controlled and sound-attenuated room (EMBLA_S7000, Embla Systems Inc., Broomfield, CO, USA). The details of this methodology have been described and used previously elsewhere [17].

The following parameters were included in the polysomnographic assessment [24]: total sleep time (TST; the amount of actual sleep time over a sleep period); sleep efficiency (TST per sleep period); sleep latency (i.e., the amount of time before starting the effective sleep period); REM sleep latency (i.e., the amount of time before starting REM sleep); and the S1 to S3 and REM sleep stages (as a percentage of TST) [25]. Arousal was defined as a ≥ 3 s increase in the electroencephalographic frequency preceded by ≥10 s of stable sleep [25]. The following data were collected for the arousal assessment: total arousal duration after falling asleep (in minutes), total number of arousals (*n*), and arousal index (number of events per hour) [25]. The apnea-hypopnea index (AHI, event count per hour of TST) was calculated as the total number of episodes of apnea and hypopnea per hour of electroencephalographically confirmed sleep. Apnea was defined as the cessation of airflow for at least 10 s combined with the effort to breathe. Hypopnea was classified as a minimum 30% reduction in thoracoabdominal movement or airflow compared with baseline lasting at least 10 s together with ≥4% oxygen desaturation [26]. Our previous study reported different polysomnographic parameters from a similar cohort [15, 17].

A board-certified physician who was unaware of the study design (and was therefore blind to patients' ophthalmic examination results) analyzed the polysomnographic tests. The polysomnographic tests were performed within 6 months of the administration of the ESS questionnaire, the ophthalmological exams, and the PLR tests. The ophthalmological exams and the PLR tests were performed within a 1-month interval.

### 2.5. Statistical Analyses

Both eyes of each participant were included in this analysis. To correct for the bias introduced by the expected correlation between the two eyes of each participant, a generalized estimating equation (GEE) was used to adjust for intereye correlations [27]. After adjusting for within-patient intereye correlations, the GEE was used to examine the relationship between the ESS score and the PLR across the entire cohort. In addition, a linear regression was used to determine the association between the ESS score and the polysomnographic parameters for both groups [27].

To obtain the correlation coefficients regarding the association between the ESS score and the PLR as well as the polysomnographic parameters, a linear regression was performed. Whenever both eyes were eligible, the right eye was arbitrarily selected for this specific analysis. Participant age was examined as a covariate.

Statistical analyses were performed using commercially available Stata software (version 13, StataCorp LP, College Station, TX, USA). The *α* level (type I error) was set at 0.05.

## 3. Results

This study examined both eyes of 40 subjects: 30 with glaucoma and 10 healthy (control group). The mean (±standard deviation) ages of the control and glaucoma groups were 56.10 ± 8.08 (range = 45 to 74) and 62.00 ± 9.39 (range = 42 to 75) years, respectively (*p* = 0.084). The demographic and clinical characteristics of the participants are summarized in Table 2. Relevant variation was present with regard to the average MD in the glaucomatous eyes, ranging from −32.28 to 0.53 dB.

### 3.1. Overall ESS Results

**Figure 1:**
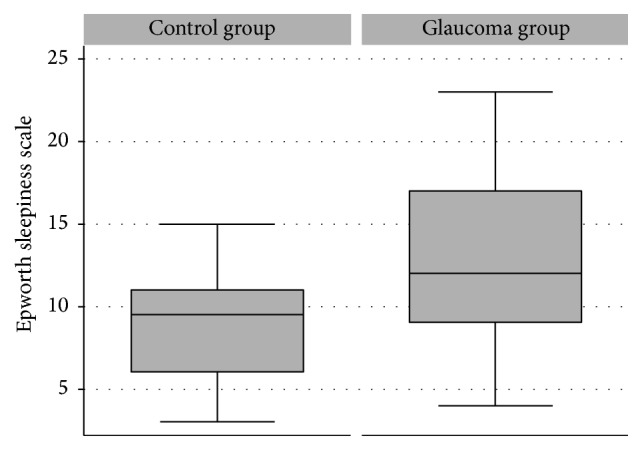
Boxplot depicting the distribution of the ESS scores in the control and glaucoma groups. Box: median and interquartile range (IQR). The whiskers show the maximal and minimal 1.5 IQR.

The mean ESS score of the glaucoma patients was significantly higher than that of the control group (13.10 ± 5.14 and 9.10 ± 3.73, resp., *p* = 0.029). With regard to sample size, nineteen glaucoma patients (63.33%) and three control patients (30.00%) reported an ESS score >10, which is considered as borderline or indicative of an increased level of chronic sleepiness; however, this difference was not significant (*p* = 0.234; Fisher's exact test). The ESS score was greater than 15 for 10 of the participants in the glaucoma group (33.33%), suggesting a high level of daytime sleepiness; however, none of the participants in the control group had an ESS score greater than 15.  Figure 1 illustrates the distribution of the ESS scores among the controls and glaucoma patients.

**Figure 2:**
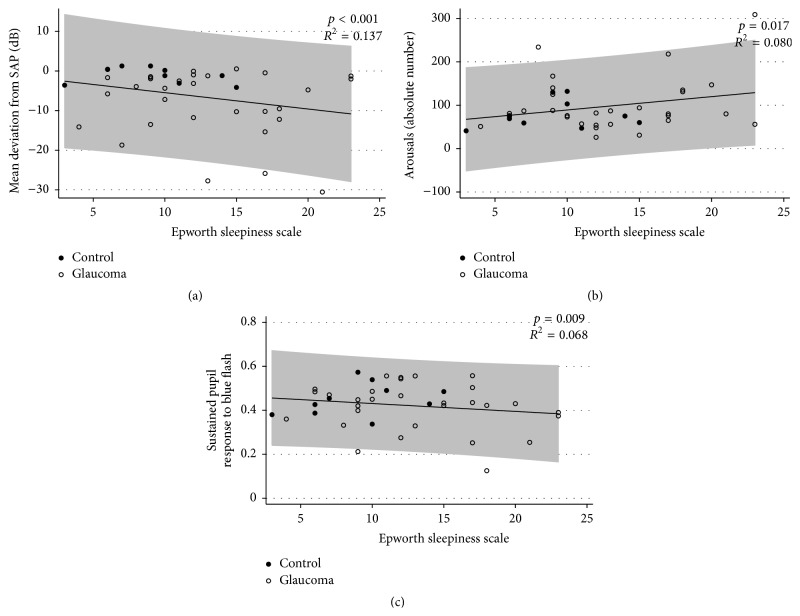
Scatterplot depicting the negative association (a) between the SAP MD and the ESS scores, the positive association between the arousal parameters at polysomnography and the ESS scores (b), and the negative relationship between the sustained pupillary response to the blue flash at 250 cd/m^2^ and the ESS score (c) in the control and glaucoma groups. The shaded area represents the prediction interval of the regression.

A significant association was found between the ESS score and glaucoma severity based on SAP MD (*p* < 0.001); specifically, higher ESS scores were related to worse damage (Figure 2(a)).

### 3.2. Association between Polysomnography and ESS Results

Table 3 shows the associations between the ESS scores and the polysomnographic parameters for the entire cohort. A significant inverse correlation was observed between the ESS score and sleep efficiency (*p* = 0.002), indicating that higher ESS scores were associated with lower sleep efficiencies. A significant association was also found between the ESS score and arousal duration after falling asleep (*p* < 0.001); specifically, higher ESS scores were related to longer arousal durations after falling asleep. The ESS score was also associated with the number of arousals and the arousal index (*p* = 0.039 and *p* = 0.013, resp.).  Figure 2(b) illustrates the association between the ESS score and the number of arousals at polysomnography for the entire cohort. Age was included in the multivariate model but did not exert a significant effect (*p* > 0.05 for all polysomnographic parameters).

### 3.3. Association between PLR Test and ESS Results

A significant inverse correlation was observed between the ESS score and the peak response to the blue flash with a luminance of 250 cd/m^2^ (*p* = 0.017, *R* ^2^ = 0.080). Furthermore, a significant correlation was found between the ESS score and the sustained response to the blue flash with luminance of 250 cd/m^2^ (*p* = 0.009, *R* ^2^ = 0.068). A higher ESS score was associated with lower peak and sustained responses to the blue flash at 250 cd/m^2^.  Figure 2(c) shows the association between the ESS score and the sustained response to the blue flash with luminance of 250 cd/m^2^ for the entire cohort.

An inverse association was also found between the ESS score and the sustained response to the red flash at 250 cd/m^2^ (10.7 log photons/cm^2^/s^2^) (*p* = 0.010; *R* ^2^ = 0.080); specifically, a higher ESS score was associated with a lower sustained response to the red flash with luminance of 250 cd/m^2^ (10.7 log photons/cm^2^/s). However, no correlation was observed between the ESS score and the peak response to the red flash at 250 cd/m^2^ (10.7 log photons/cm^2^/s; *p* = 0.199; *R* ^2^ = 0.021).

In addition, age was examined in the multivariate model; however, it was found not to exert a significant effect on the associations between the ESS score and either the peak or sustained response to the blue flash with luminance of 250 cd/m^2^ for either the control or glaucoma group (*p* = 0.403 and *p* = 0.290, resp.). In addition, age did not significantly affect the associations between the ESS score and either the peak or sustained response to the red flash with luminance of 250 cd/m^2^ for either the control or glaucoma group (*p* = 0.223 and *p* = 0.375, resp.).

## 4. Discussion

This study used the ESS to evaluate sleep disorders, specifically daytime sleepiness, in glaucoma patients. In addition, we addressed the relationship between the ESS score and both polysomnographic measures and PLR in glaucoma patients. Increased daytime sleepiness, as measured by the ESS, was associated with arousal duration after falling asleep, sleep efficiency, number of arousals, and the arousal index at polysomnography. In addition, increased ESS scores were associated with reduced peak and sustained PLRs to a blue flash with luminance of 250 cd/m^2^. To the best of our knowledge, this study is the first to identify the associations between daytime sleepiness and polysomnography and PLR parameters. Sleep disorders (evaluated using ESS and polysomnography) and PLR might be related to certain types of ipRGCs [29].

The results of this study are in agreement with those of previous studies showing that glaucoma is associated with a decrease in sleep quality [16, 30, 31]. Wang et al. used the PSQI to investigate whether glaucoma affects sleep quality and to evaluate the differences between disparate types of glaucoma (i.e., primary open-angle glaucoma and primary closed-angle glaucoma) [16]. These authors found that the prevalence of sleep disorders was higher among patients with glaucoma and that the patients with primary closed-angle glaucoma had a greater incidence of sleep disorders than those with primary open-angle glaucoma [16]. Using the ESS, we demonstrated that patients with glaucoma showed increased daytime sleepiness. The ESS is a self-report questionnaire that is considered as the gold standard for assessing daytime sleepiness [18]; however, to date, the ESS score has never been used to investigate glaucoma patients. We hypothesized that ocular damage in patients with glaucoma affects all types of RGCs (including ipRGCs) and that this damage is linked to the dysregulation of the circadian system, thereby leading to decreased sleep quality and resulting in excessive daytime sleepiness.

The finding in our study supported these hypotheses, showing that patients with glaucoma have higher daytime sleepiness associated with worse parameters in polysomnography. The excessive daytime sleepiness observed among glaucoma patients was likely because of the increased number or total duration of arousals after falling asleep, decreased sleep efficiency, and briefer TST. Daytime sleepiness (as measured using the ESS) was also associated with different polysomnographic parameters such as sleep efficiency, arousal duration after falling asleep, arousals, and the arousal index. A previous study performed by our group demonstrated that patients with glaucoma have significantly lower average TSTs and sleep efficiencies as well as longer arousal durations after falling asleep [17].

Daytime sleepiness was also associated with PLR, a well-known indicator of the afferent input from the retina and the optic nerve. Most optic neuropathies show an association between PLR and disease severity [32–36]. Briefly, the PLR shows a transient phase of pupillary constriction, which is attributed to rod and cone input to the ipRGCs, followed by sustained pupil constriction, which is primarily driven by the melanopsin response in the ipRGCs [20, 37]. Previous studies have shown an inverse correlation between sustained pupillary constriction and glaucoma severity [11, 13, 15]. For example, Gracitelli et al. showed that the sustained response to the blue flash at 250 cd/m^2^ (which preferentially stimulates ipRGCs [3, 4]) was reduced in patients with advanced glaucoma (i.e., patients with SAP MD worse than −12 dB) [15]. In addition, a thinner RNFL was associated with a reduced sustained pupillary response [15]. The present study adds new information by showing that, in addition to the decreased sustained pupillary response in glaucoma patients, the degree of daytime sleepiness is associated with a reduced sustained response to a blue flash at 250 cd/m^2^. One possible explanation for this finding is that ipRGCs are among the cells in the total RGC population that are damaged in glaucoma patients; thus, they affect several functions of ipRGCs, including the PLR and circadian rhythms. Importantly, although a significant association was observed between daytime sleepiness and the PLR in this study, the correlation coefficient was not substantial (*R* ^2^ = 0.080 for the correlation between the ESS score and peak PLR and *R* ^2^ = 0.068 for the correlation between the ESS score and the sustained PLR). Thus, important, unknown factors most likely contribute to this correlation. For example, the subjectivity of all self-report questionnaires might affect the final score. In addition, studies performed by Pack et al. and Gooneratne et al. showed that self-reported questionnaire of sleepiness can often be inaccurate when viewed alongside quantitative measures of alertness and sometimes is often viewed as a common and natural aspect of aging that can lead to a misinterpretation of the results [38, 39].

In addition, daytime sleepiness, in glaucoma group, was associated with a sustained PLR to the red flash. The peak and sustained PLRs to a red flash are well known to be correlated with the number and function of the rods and cones, not ipRGCs. One hypothesis explaining this finding is that rod and cones photoreceptors might also be affected in patients with chronic or advanced glaucoma, resulting in decreased signaling from these cells [40–42]. However, this topic remains controversial in the literature [40–43], and additional studies must be conducted to clarify the actual connection between the functions of ipRGCs and rod and cones photoreceptors.

Another interesting issue is the controversy concerning the relative preservation of ipRGCs among the diversity of RGCs [21, 44–47]. Li et al. showed that although the conventional RGC number was decreased in rats with increased IOP, the number of ipRGCs did not change. This result suggests that ipRGCs were resistant to the deleterious effects caused by increased IOP [45]. However, another study in mice demonstrated that ipRGCs appeared to be resistant to damage resulting from IOP elevation at an early age (approximately 5 months) but became vulnerable at a later age (approximately 11 months) [48]. The authors of that study suggested that the magnitude of IOP elevation required to damage ipRGCs must be greater than that required to induce RGC damage [48, 49]. In addition, Drouyer et al. and de Zavalía et al. showed that glaucomatous rats and other rodents exhibited a delayed phase angle with respect to darkness [46, 47]. These results are in agreement with ours, suggesting that glaucoma induces a circadian system dysfunction. One explanation for the differences between certain animal models and studies of patients with glaucoma is the endpoint evaluated, that is, morphological versusfunctional endpoints [49]. The primary focus of human studies has typically been ipRGC function, whereas the number and morphology of these cells have usually been considered in animal studies. The resistance mechanism that underlies the survival of ipRGCs is currently unknown. Although this question persists, our study analyzed two different functions involving ipRGCs in the same group of patients, and we found a strong association between them. Thus, our results strongly suggest that both non-image-forming functions of ipRGCs were affected.

Recently, Chen et al. demonstrated that there is not one specific type of ipRGC; rather, different subpopulations of ipRGCs exist with similar properties that innervate disparate brain regions to execute specific functions, such as the PLR and circadian photoentrainment [50]. In this study, pupil constriction, circadian oscillation (wheel running activity), the adjustment of the circadian clock to different light stimuli (i.e., circadian photoentrainment, “jet-lag” paradigms, phase shifting, and skeleton photoperiod), and the direct effect of constant and ultradian light on activity were measured [50]. The results showed that ipRGCs comprise functionally distinct subpopulations that differentially express a specific transcription factor [50]. ipRGCs that do not express this transcription factor innervate the suprachiasmatic nucleus of the hypothalamus, whereas ipRGCs that express this transcription factor innervate other known brain targets such as the olivary pretectal nucleus. Thus, the ipRGCs that express this transcription factor are associated with the PLR but not with circadian photoentrainment [50]. Because we did not evaluate this issue in the present study, future investigations should be conducted to elucidate the subpopulations of ipRGCs to determine how they are involved in non-image-forming functions.

The primary clinical finding of this study was that glaucoma patients had increased daytime sleepiness compared with healthy participants, and these results were associated with worse polysomnographic parameters and lower sustained PLRs to a blue flash with luminance of 250 cd/m^2^. Therefore, circadian rhythm regulation, which is linked with ipRGCs' functions, should be evaluated in certain patients with glaucoma. Excessive daytime sleepiness is well known to affect quality of life, daytime function, and mortality; furthermore, previous studies of geriatric populations have revealed that excessive daytime sleepiness is associated with age, disability, dementia, and vision and hearing impairments [51].

This study has limitations. First, a relatively small number of patients were included in this study. Although future studies with greater numbers of patients should be undertaken to better understand the role of ipRGCs in sleep disorders, the present study did show strong agreement between the subjective and objective measures of sleep quality among patients with glaucoma. Second, sleep patterns are most likely affected by factors other than the hypothesized ipRGC loss associated with glaucoma. However, the subjective and objective measures of sleep quality (i.e., the ESS questionnaire and the polysomnographic parameters) strongly suggested that the damage to ipRGCs associated with glaucoma results in certain types of sleep disorders. Furthermore, regarding the primary endpoint of this study, we relied on self-reported histories of daytime sleepiness; it is possible that inaccuracies were present in patient recollection and reporting. However, we also used polysomnographic parameters (i.e., the gold standard test of sleep quality), and these parameters were in agreement with the ESS scores. In addition, because patient age is associated with a decrease in sleep quality and can affect PLR, this variable might have affected our final results [16, 51]. Although the influence of age-related factors could not be completely excluded, our study did not reveal any significant differences in age between the two groups, and the multivariate analysis found that age was not a significant covariate. In addition, previous studies have shown that the postillumination response did not significantly decrease with age [52] and that only pupil diameter was reduced among the elderly population [52].

In conclusion, the results of this study demonstrated that patients with glaucoma had increased daytime sleepiness relative to healthy controls as measured by a self-report questionnaire. In addition, daytime sleepiness was associated with a reduced sustained pupillary response and polysomnographic parameters. These ipRGC functions might be impaired in patients with glaucoma, thereby affecting their quality of life.

## Figures and Tables

**Table 1:** Photometric measures for different photoreceptors' inputs to circadian and neurophysiological light responses in humans [2].

Photoreceptor	Photopigment	Spectral sensitivity function	Unit of measure^a^
Short-wavelength (S) cones	S-cone photopsin (cyanolabe)	Cyanolabe response function *N*_sc_ (*λ*)	Cyanopic illuminance (cyanopic-lux)
Medium-wavelength (M) cones	M-cone photopsin (chlorolabe)	Chlorolabe response function *N*_mc_ (*λ*)	Chlorolabe illuminance (chloropic-lux)
Long-wavelength (L) cones	L-cone photopsin (erythrolabe)	Erythrolabe response function *N*_lc_ (*λ*)	Erythrolabe illuminance (erythropic-lux)
ipRGCs (intrinsic photosensitivity)	Melanopsin	Melanopsin response function *N*_*z*_ (*λ*)	Melanopsin illuminance (melanopic-lux)
Rods	Rod opsin	Rod opsin response function *N*_*r*_ (*λ*)	Rhodopic illuminance (rhodopic-lux)

a = each unit of measure (Ea, where a specifies the retinal photopigment) is derived by convoluting the spectral power distribution of incident light (Eel) with the relevant spectral sensitivity function, which in turn is defined by the photopigment spectral sensitivity adjusted for prereceptoral filtering in a standard observer (Na(l); see the online reference (http://www.cie.co.at/index.php?i_ca_id=983) [28] for full functions and a detailed description of their derivation) according to the equation Ea = 72 983.25 R Eel(l) Na(l) dl. Species-specific variants of the spectral sensitivity functions may be required for nonhuman applications to account for differences in prereceptoral filtering and photopigment spectral sensitivity [2].

**Table 2:** Demographic and clinical characteristics (mean ± standard deviation [median, interquartile range]) of control and glaucoma participants.

	Control group (*N* = 20 eyes of 10 subjects)	Glaucoma group (*N* = 60 eyes of 30 subjects)	*p* value
Age, years	56.10 ± 8.08 (55.50, 54.00 to 58.00)	62.00 ± 9.39 (64.50, 53.00 to 70.00)	0.084^a^
Female, %	8 (80%)	20 (67%)	0.693^c^
Ancestry, %			0.401^c^
Caucasian	9 (90%)	21 (70%)	
African-American	1 (10%)	9 (30%)	
MD worse eye, dB	−1.28 ± 2.03 (−1.23, −3.10 to 0.44)	−11.82 ± 10.68 (−7.15, −20.22 to −3.07)	**<**0.001^b^
MD better eye, dB	−0.04 ± 1.60 (0.27, −1.18 to 1.52)	−7.00 ± 7.97 (−2.90, −11.78 to −1.34)	**<**0.001^b^
Average IOP, mmHg	13.42 ± 1.87 (13.00, 12.00 to 14.00)	16.90 ± 2.82 (16.00, 15.00 to 18.00)	**<**0.001^b^
Cup/disc ratio	0.34 ± 0.10 (0.30, 0.30 to 0.40)	0.80 ± 0.15 (0.80, 0.70 to 1.00)	**<**0.001^b^
CCT, *µ*m	539.95 ± 30.24 (534.00, 519.00 to 562.00)	521.76 ± 34.52 (520.50, 498.00 to 547.00)	0.069^a^
Visual acuity, logMAR	0.04 ± 0.07 (0.00, 0.00 to 0.10)	0.11 ± 0.09 (0.10, 0.00 to 0.20)	**<**0.001^a^

MD = mean deviation; dB = decibels; IOP = intraocular pressure; mmHg = millimeters of mercury; CCT = central corneal thickness; logMAR = logarithm of the minimum angle of resolution.

^a^Student's *t*-test; ^b^Wilcoxon's rank-sum test; ^c^Fisher's exact test.

Significant values are in boldface.

**Table 3:** Associations (*R* ^2^) between the polysomnographic parameters and the ESS scores^*∗*^.

Parameter	*p* value	*R* ^2^
Arousal duration after falling asleep (min)	**<0.001**	0.252
Sleep efficiency (%)	**0.002**	0.214
Arousals (*n*)	**0.039**	0.163
Arousal index (*n*/h)	**0.013**	0.133
TST (min)	0.050	0.159
Sleep latency (min)	0.592	0.120
REM sleep latency (min)	0.667	0.119
Sleep stage (%)		
S1	0.848	0.118
S2	0.057	0.157
S3	0.150	0.140
REM	0.782	0.118

^*∗*^Linear regression model including the polysomnographic parameters and the ESS scores.

Significant values are in boldface.
